# CT Evaluation of Swallowed Foreign Bodies Located in the Gastrointestinal System

**DOI:** 10.7759/cureus.26355

**Published:** 2022-06-26

**Authors:** Muhammed Akif Deniz, Mehmet Turmak

**Affiliations:** 1 Radiology, Dicle University School of Medicine, Diyarbakır, TUR

**Keywords:** radiology, perforation, gastrointestinal system, foreign body, computed tomography

## Abstract

Background and objective

CT imaging is important in detecting the location and the structure of swallowed foreign bodies and assessing their complications, due to its features such as the ability to show the detailed anatomical structure and enable multiplanar examination. In this study, we aimed to analyze the CT findings of swallowed foreign bodies located in the gastrointestinal tract and compare them with the data in the existing literature.

Materials and methods

We conducted a retrospective archive study to achieve our goals. Patients who presented to our radiology department with the preliminary diagnosis of foreign body ingestion, abdominal pain, or acute abdomen and were found to have a foreign body in the gastrointestinal tract on CT between April 2018 and April 2021, especially those in whom the presence of a foreign body was confirmed by endoscopy or surgery, were included in our study. The patients were evaluated in terms of age, gender, foreign body type, foreign body localization, and complications.

Results

A total of 31 patients (15 males and 16 females) were included in the study. The patients’ age ranged from 1 to 67 years, and the mean age was 28.5 ±5.4 years. The most common foreign bodies found were metallic toy parts (n=11, 35%), and most of the foreign bodies were located in the ileum (n=9, 29%) of the patients. Complications were observed in nine patients (29%). In patients with complications, the most common finding was perforation (n=3, 38%).

Conclusion

When a CT exam is performed on patients with abdominal pain, the gastrointestinal tract should be carefully evaluated to determine if a foreign body is involved and to analyze the complications caused by the foreign body.

## Introduction

Ingestion of foreign bodies is commonly encountered in children. Although rarely seen in adults, its rate of mortality and morbidity among adults is much higher [1]. About 80-85% of ingested foreign bodies move from the stomach to the intestines and are excreted by passage without causing any symptoms or harming the body, while the rest require treatment [2]. The size, type, structure, and number of the swallowed bodies are extremely important in treatment planning [3].

Direct radiography is a frequently used examination method in radiological imaging and in the follow-up of patients with ingested foreign bodies due to its advantages such as low cost, easy access, and relatively low ionizing radiation. However, direct radiography may be insufficient in foreign body localization and complications. CT is important in detecting the location and the structure of the foreign body and examining its complications, due to its features such as showing the detailed anatomical structure and enabling multiplanar examination [1-4].

In this study, we discuss the CT findings of swallowed foreign bodies located in the gastrointestinal tract and compare them with the relevant literature.

## Materials and methods

This was a retrospective archive study. Patients who presented to our radiology department with the preliminary diagnosis of foreign body ingestion, abdominal pain, or acute abdomen and were found to have a foreign body in the gastrointestinal tract on CT between April 2018 and April 2021, especially those in whom the presence of a foreign body was confirmed by endoscopy or surgery, were included in our study.

The patients were evaluated in terms of age, gender, foreign body type, foreign body localization, and complications. Complication types and complication rates according to age were examined in patients with complications.

Abdominal pelvic CT scans of our patients included in the study were performed with 64 detector multislice Philips Brilliance V2.6.1 (2007, Philips Healthcare, Eindhoven, Netherlands) and 16 detector Toshiba Activion V3.00 (2010, Canon Medical Systems Corporation, Ōtawara, Japan) devices. Cross-sectional images were taken from the liver dome to the pelvic region that was at least in the 3-mm range of section. The images were evaluated in the workstation (Philips Extended Brilliance Workspace, Philips Healthcare). Images were first analyzed in axial sections. Later, sagittal and coronal sections were also used by creating 3D reformatted images; 1-1.5 cc IV contrast material per kg was given to patients who underwent contrast-enhanced imaging.

Patients who received a preliminary foreign body ingestion diagnosis but did not have a CT scan or had a CT scan that did not detect a foreign body, and patients with a foreign body detected on CT but who did not have surgical or endoscopic intervention and thus the foreign body appearance was not confirmed in terms of intervention, were excluded from the study. In addition, since our study focused on patients who swallowed foreign bodies, patients with foreign bodies (scatter pellets, firearm bullets, sharp objects, etc.) in the gastrointestinal tract as a result of penetrating trauma were also excluded from the study.

The ethical approval for the study was obtained from the Non-Invasive Clinical Research Ethics Committee, Dicle University Faculty of Medicine (date: 09.01.2020, number: 89).

The SPSS Statistics software package (IBM Corp., Armonk, NY) was used to analyze the data. Mean and standard deviations were used to present age, gender, foreign body type, location, and complication parameters as a statistical method in our study. Conformity to the normal distribution was checked, and the independent samples t-test was used for pairwise comparisons for those data with normal distribution. The chi-square test was used in the examination of age-related complications.

## Results

**Figure 1:**
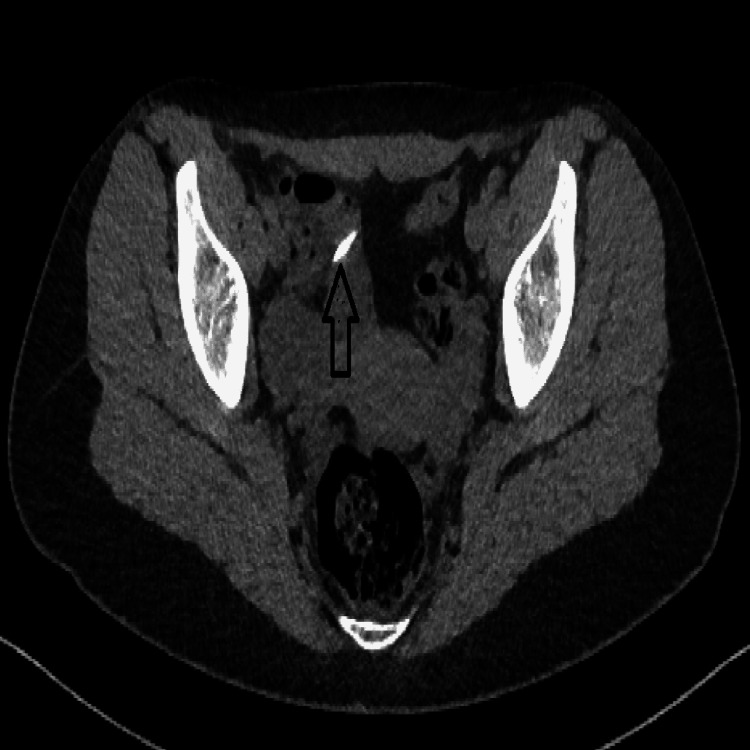
15-year-old female patient: needle (arrow) found at ileum level

**Figure 2:**
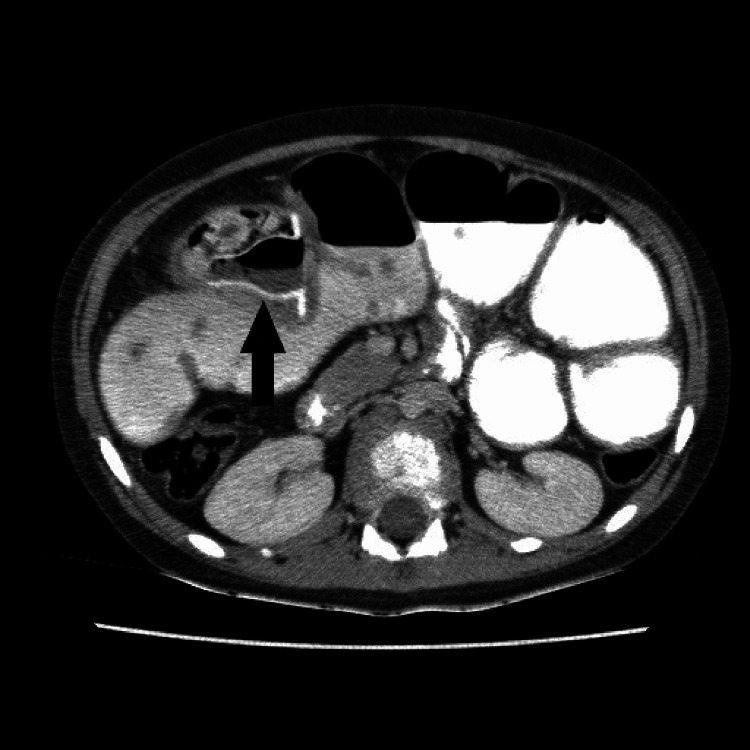
10-year-old female patient: baby bottle cap at the level of the jejunum (arrow) and secondary ileus appearance

**Figure 3:**
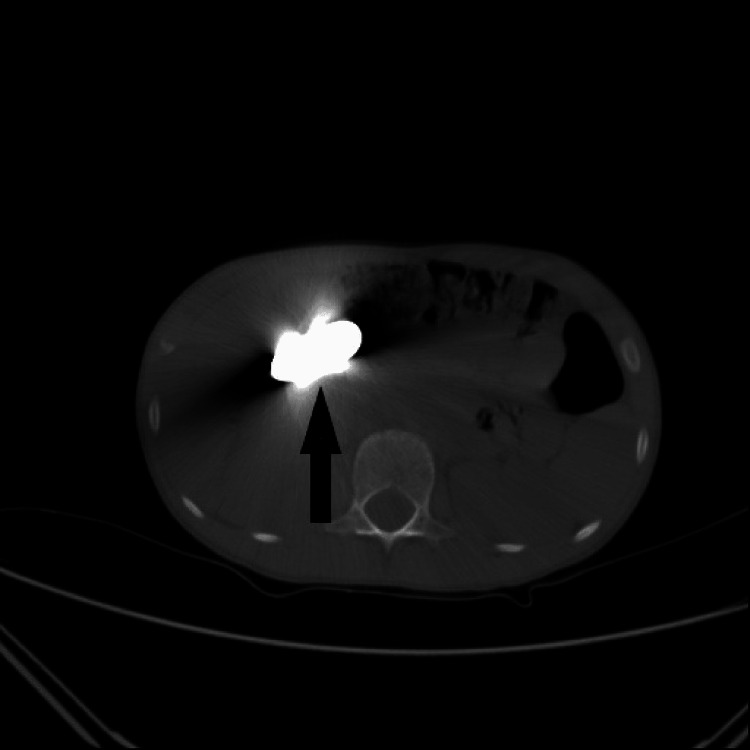
12-year-old male patient: two magnets (arrow) at duodenum level

A total of 31 patients (15 males and 16 females) were included in the study. The patients’ age ranged from 1 to 67 years, and the mean age was 28.5 ±5.4 years. An abdominal CT scan was performed in 20 patients with contrast and one without contrast was performed in 11. Foreign bodies were located in the ileum (Figure 1) of nine patients, in the duodenum of six, in the stomach of five, in the cecum of four, in the ascending colon of three patients, and in the transverse colon, sigmoid colon, jejunum (Figure 2), and anal canal of one patient each. The foreign bodies found were metallic toy parts in 11 patients, pins in nine patients, wooden toothpick pieces in six patients, magnets in one patient (Figure 3), baby bottle cap in one patient, whole bone fragments in two patients, and walnut with shell in one patient (Table 1).

**Table 1:** Demographic characteristics and CT findings of the cases CT: computed tomography; SD: standard deviation; SMV: superior mesenteric vein

		Count	Percentage
Age (years), mean ±SD: 28.5 ±5.4			
Gender	Male	15	48
	Female	16	52
CT	With contrast	20	64
	Without contrast	11	36
Foreign body location	Ileum	9	28
	Duodenum	6	20
	Stomach	5	16
	Cecum	4	13
	Ascending colon	3	10
	Transverse colon	1	3
	Sigmoid colon	1	3
	Jejunum	1	3
	Anal canal	1	3
Types of foreign bodies	Toy/metallic toy piece	11	36
	Pin	9	28
	Toothpick wood pieces	6	20
	Whole bone	2	6
	Magnet	1	3
	Bottle cap	1	3
	Whole-shell walnut	1	3
Complications	Present	9	28
	Absent	22	71
Complication type	Perforation	3	10
	Wall thickening - increase in mesenteric density	2	6
	Ileus	2	6
	Ureteral fistulization	1	3
	SMV thrombosis	1	3

**Figure 4:**
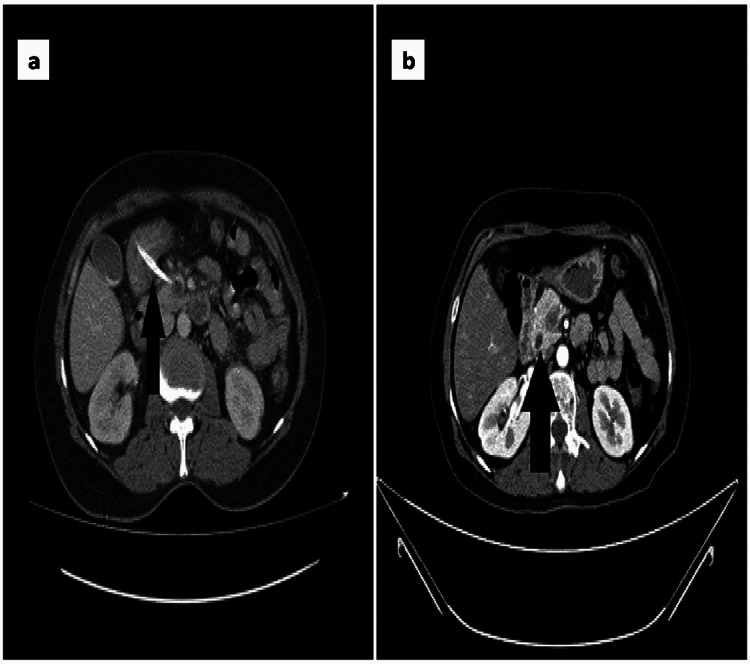
37-year-old female patient: (a) stomach-located bone fragment (arrow) and its extension to SMV, (b) thrombus appearance in SMV (arrow) two weeks after foreign body removal SMV: superior mesenteric vein

Complications were observed in nine patients (29%). In patients with complications, the most common finding was perforation in three patients (38%), foreign body reaction in the form of wall thickening-increased mesenteric density in two patients (25%), ileus in two (25%) patients (Figure 1), ureter fistulization in one (12.5%) patient, and superior mesenteric vein (SMV) thrombosis in one (12.5%) patient (Figure 4).

Foreign body localization and perforation were at the level of the ileum in all cases with perforation. Cases with ileus had a whole-shell walnut located in the ileum and a bottle cap located in the jejunum.

Of the 31 patients in our study, 10 (32%) were children (under 16 years old), and 21 (68%) were in the adult age group. Complications were observed in two (20%) of 10 pediatric patients and seven (33%) of 21 adult patients. The two pediatric patients with complications had ileus as a result of swallowing a bottle cap and perforation as a result of needle swallowing. Of the seven adult patients with complications, two had perforation, two had a foreign body reaction in the form of wall thickening-increased mesenteric density (Figure 2), one had ileus, one had SMV thrombosis, and one had ureteral fistulization. When the age groups and the frequency of complications were compared, no significant difference was found between pediatric and adult patients (p=0.677) (Table 2).

**Table 2:** Association in terms of age-related complications *Chi-square analysis was performed

		With complication		Without complication		P-value*
		Count	%	Count	%	
Age group	Pediatric	2	20.0	8	80.0	0.677
	Adult	7	33.3	14	66.7	

## Discussion

Complications were observed in approximately 29% of the patients in our study in which swallowed foreign bodies in the gastrointestinal tract were evaluated with CT. We also determined that 63% of the patients with complications were in the adult age group. While most of the foreign bodies located in the gastrointestinal tract are excreted spontaneously, a few (10-15%) require intervention due to complications. The higher complication rate in our study compared to the literature could be attributed to the fact that wall thickening and mesenteric density increases were accepted as complications in our study. This group comprised approximately 25% of the group with complications. Although foreign body ingestion can occur at any age, it is much more common in children. In adults, it is more common among the elderly, individuals with intellectual disabilities, and alcoholics. Also, accidental or inadvertent swallowing is also common in adults. Although foreign body ingestion is less common in the adult age group, the mortality and morbidity rate is higher [1,5].

Of the patients in our study, 32% were children (under 16 years old) and 68% were adults. Regarding pediatric patients, admission occurred when the patients themselves or their relatives reported ingestion of a foreign body or when the relatives suspected foreign body ingestion. Although the preliminary diagnosis of foreign body ingestion was reported in 12 patients in the adult age group, in nine patients, it was acute abdomen or abdominal pain. When nine adult patients with a prediagnosis of an acute abdomen or abdominal pain were evaluated, a mass was diagnosed in two patients (on suspicion in an eccentric ultrasonographic examination), acute cholecystitis in three patients, and perforation in four patients. After the foreign body was detected by CT in nine patients in this group, four patients remembered swallowing a foreign body while five of them did not remember swallowing any foreign body. Foreign bodies were mostly in the form of wooden toothpick pieces in patients who did not remember swallowing a foreign body. We think that these foreign bodies were swallowed unconsciously with food intake. Also, complications were observed in 20% of pediatric patients and 33% of adult patients. Fewer complications observed in pediatric patients can be attributed to the fact that the prediagnosis and diagnosis rates of foreign body ingestion in pediatric patients are higher than in adult patients, as well as the delay in admission to the hospital in adults and the higher rate of ingestion of pointed and sharp-edged objects, which have a higher potential for complications, in adults.

Some studies in the literature have reported that 80% of swallowed foreign bodies are radiopaque, which can be seen in 88% of the radiographs including those of the neck and thorax [2,6-9]. The first radiological imaging employed for the detection of a swallowed foreign body is direct radiography. Radiopaque foreign bodies are seen directly on the radiograph. However, direct radiography may be insufficient for the detection of the structure and localization of the foreign body and the detection of complications related to the foreign body.

CT is frequently used in the diagnosis and treatment planning of foreign body ingestion. The sensitivity and specificity of CT were found to be in the range of 94 and 100%, respectively. CT is extremely sensitive in terms of the structure, number, shape, localization, and complications of the swallowed foreign body. In addition, CT is extremely sensitive in the detection of foreign bodies that are not radiopaque and cannot be detected on direct radiography [5-10]. Although IV contrast material is not recommended for primary foreign body detection, it is important in terms of intra-abdominal inflammatory processes and differential diagnosis. Oral contrast agent use is not recommended as it may hide the foreign body [1-3].

In our study, CT without contrast was performed in 11 patients, and CT with IV contrast was performed in 20 patients. The high number of contrast-enhanced CT in our study could be due to the lack of a preliminary diagnosis of foreign body ingestion in all patients in our study and the suspicion of acute abdomen findings in these patients. In addition, we think that contrast-enhanced CT scans were performed to investigate the presence of complications in patients with a preliminary diagnosis of foreign body ingestion. In pregnant and pediatric age groups, low-dose use is recommended in cases where CT is indicated [1-3]. There were no pregnant patients in our study. Low-dose protocols created by our unit were used for CT scans of pediatric patients in our study.

Foreign bodies in the upper gastrointestinal tract are most frequently observed in the esophagus and stomach. Foreign bodies are frequently seen in the lower gastrointestinal tract at the level of the ileum, where the lumen is relatively narrow [6,7,11-13]. In our study, foreign bodies were most frequently observed in the ileum, duodenum, and stomach, which is compatible with the findings in the literature. In our study, no foreign bodies were detected in the esophagus. This could be due to the fact that foreign bodies located in the esophagus are easily detected by direct radiography and there is no need for CT.

Foreign bodies may be classified into organic or inorganic according to their structure and features, and blunt or sharp based on their traumatic properties. The size of foreign bodies is important in treatment planning. Due to the curvature of the duodenum and narrowness at the level of the Treitz ligament, long objects are likely to become stuck at these levels [1-4,14-18]. Objects larger than 2.5 cm at the stomach level are unlikely to pass through the pylorus. In addition, stenosis at the level of the ileocecal valve may prevent the passage of foreign bodies. The immobilization of sharp or pointed objects longer than 4 cm and wider than 2 cm in the stomach or duodenal region for more than three days and the persistence of blunt objects for more than seven days require endoscopic or surgical removal. For all these reasons, the size of the foreign body should be measured in more than one plane and reported to the clinician. Many types of foreign bodies have been described in the literature. The most common radiopaque substances are bones, coins, pins, and batteries, while the most common radiolucent substances are fish and chicken bones, wood, plastic, and thin metal objects [1,3,8-10]. The most common foreign bodies detected in our study were toys/toy parts in the pediatric age group and pins in the adult age group. It is common for women who wear turbans to hold the attached needle between their teeth and accidentally swallow it [11]. It was determined that 30% of the patients in our study had this type of needle-swallowing history.

Ingested foreign bodies are frequently excreted naturally from the gastrointestinal tract without any complications [1,12-15]. However, some complications may occur depending on the type of the foreign body, its characteristics (pointed, blunt tip), removal duration, and the number of objects. In these cases, various gastrointestinal system complications may be observed, including perforation, bleeding, ileus, fistulization, intussusception, and even sepsis [2,4-5]. Perforations are most commonly seen in the duodenum, ileocecal region, and rectosigmoid. Foreign body detection is very important in terms of addressing all these complications. Sepsis and massive bleeding can be observed in patients who do not receive treatment [2,4]. In our study, complications were detected at a rate of 29%, and the most common (44%) was at the ileum level. In patients with complications, the most common finding was perforation (38%), followed by wall thickening-mesenteric density increase in 25%, ileus in 25%, ureteral fistulization in 12.5%, and SMV thrombosis in 12.5%. Sharp and pointed objects are associated with a greater risk of perforation. In addition, the extension of such foreign bodies to surrounding tissues and adjacent vascular structures has been reported [1-5].

In our study, the swallowed foreign body was sharp and pointed in 88% of the nine patients with complications. Also, there were two complications that were not reported previously in the literature. The first of these was the extension of the needle located in the colon to the ureter and its fistulization in this area, and the other was the significant extension of the bone located in the stomach to the SMV and its intravascular placement. In the latter case, SMV thrombosis persisted for a while after the removal of the foreign body.

The main limitations of our study are the relatively small sample size, the difference in the number of patients between the pediatric and adult age groups, the absence of patients with foreign bodies detected in the esophagus, and the fact that a patient group was not included in the study because CT scan was not performed in them.

## Conclusions

Early diagnosis and intervention are very important in reducing morbidity and mortality in patients who swallow foreign bodies. When a CT exam is performed in patients with abdominal pain, the gastrointestinal tract should be carefully evaluated to determine if a foreign body is involved and to assess the complications caused by the foreign body. CT imaging can help with the direct visualization of the swallowed foreign body, and the size, structure, and localization of the foreign body can be well defined. Knowledge of these parameters is very important in the management of ingested foreign bodies. Timely implementation of appropriate treatment strategies can be facilitated by the radiologist reporting the salient radiographic and imaging features of foreign bodies in detail.
